# The Thirty-Fourth Annual Meeting of the Mississippi Valley Association of Dental Surgeons

**Published:** 1878-05

**Authors:** Edward G. Betty


### THE THIRTY-FOURTH ANNUAL MEETING OF THE MISSISSIPPI VALLEY ASSOCIATION OF DENTAL SURGEONS.

The association convened according to adjournment at ten o'clock a. m., on Wednesday, March 6th, 1878, with President Rawls in the chair. Prayer by Dr. Leslie. Owing to the absence of the secretary, E. G. Betty was elected secretary pro tem. Minutes of previous meeting were read and approved. The executive committee reported the following programme for discussion:

First. Dental hygiene.

Second. Dental medication; local and general.

Third. Affections accompanying first dentition.

Fourth. Artificial dentures.

Fifth. Operative dentistry.

Sixth. Chemical processes that occur in the mouth and its fluids.

Seventh. Dental education and professional culture.

Eighth. Suppuration of the gums and its treatment.

On motion of Dr. J. Taft, it was resolved to hold the session from 9 a. m. to 12:30 p. m.; from 2 p. m. to 4:30 p. m. and from 7:30 p. m. till adjournment.

The discussion of the first subject, dental hygiene, being in in order was taken up and opened by Dr. J. Taft, followed by Drs. Meredith, Osmond, H. A. Smith, Berry, G. W. Keely and H. M. Reid.

### AFTERNOON SESSION.

The association was called to order at two o'clock by President Rawls. On motion it was decided to have the minutes read upon the opening of each morning session, instead of at every session. By vote of the society, visiting dentists were accorded the privileges of the floor.

The first subject was resumed and the discussion continued by Drs. J. Taft, Harriman, Ulrey and Leslie. The committee on membership reported the names of S. J. Way, of Waynesville, O.; W. S. Rawls, of Connersville, Ind.; A.

W. Smith, of Richmond, Ky. and W. H. Sillito, of Xenia, O., for election to membership, all of whom were elected. The treasurer, Dr. Cameron read his report. On motion of Dr. H. A. Smith, it was resolved to combine the first and third subjects and discuss them in conjunction.

Adjourned till nine a. m., Thursday, on account of the commencement exercises of the Ohio Dental College.

MARCH 7TH, MORNING SESSION.

Society called to order by President Rawls at nine o'clock. Minutes read and approved. A letter from Dr. W. H. Rehwinkle was read, in which he regretted his inability to be present. The first subject was passed, and the discussion of the second was taken up and opened by Dr. Hunter, followed by Drs. J. Taft, Reid, H. A. Smith and others. It was moved and adopted that Dr. C. S. Smith, be allowed the first half hour of the afternoon session to exhibit and explain matters pertaining to celluloid, On motion it was resolved to adjourn at twelve o'clock. After some further discussion on dental medication the society adjourned.

AFTERNOON SESSION.

Meeting called to order at two o'clock by President Rawls. The hour having arrived which was set for an exposition on celluloid by Dr. C. S. Smith, that gentleman took the floor and held the attention of the members by his interesting and instructive remarks.

The fourth subject on the programme, artificial dentures, was announced, being opened by Dr. VanAntwerp, and discussed by Drs. Osmond, Hall, Smith and Keely, with others. The committee on membership reported the names of the following gentlemen for membership: Drs. A. T. Good, A. Taft and W. S. Howe, who were duly elected.

EVENING SESSION.

Meeting called to order by First Vice-President Hunter The recording secretary being absent, W. H. Sillito was elected to fill the vacancy, The fifth subject, operative dentistry was opened by Dr. J. Taft, followed by Drs. Keely, C. S. Smith, Osmond, H. A. Smith, Hunter, Canine, Berry and Meredith. Adjourned.

MARCH 8TH, MORNING SESSION.

The society convened at 9:30 a. m., with Vice-President Hunter in the chair. On motion it was decided to proceed with the election of officers at 11:30 o'clock. Drs. Wm. Taft and F. Sage were appointed to nominate delegates to the American Dental Association. The subject of operative dentistry was continued by Drs. Sage, J. Taft, Leslie and others. On motion this subject was passed, and instead of following the regular order the sixth and seventh subjects were dropped, and the eighth, suppuration of the gums, was taken up and partially discussed.

ELECTION OF OFFICERS.

Dr. F. A. Hunter, President; Dr. J. S. Cassidy, First Vice-President; J. W. Jay, Second Vice-President; Dr. E. G. Betty, Recording Secretary; Dr. C. I. Keely, Corresponding Secretary; Dr. J. G. Cameron, Treasurer.

The committee appointed to nominate delegates to the American Dental Association reported the following names: A. T. Good, S. J. Way, N. S. Hoff, H. M. Reid, J. A. Stipp, A. F. Emminger, J. S. Rice, F. W. Sage, W. S. Howe, A. W. Smith, E. G. Betty, J. W. Jay, C. R. Taft, Chas. Welch, F. H. N. Smith, S. M. Cummings, all of whom were elected,

On motion it was decided to have blank certificates printed for delegates.

The President elect was conducted to the chair by Drs. H. A. Smith and S. B. Browne. Dr. J. Taft moved a vote of thanks to the retiring officers which was unanimously carried.

The President announced the following standing committees: Drs. A. O. Rawles, L. P. Meredith and D. W. Clancy, Executive. Drs. N. S. Hoff, H. M. Reid and J. T. McMillan, Membership. Drs. J. Taft, H. A. Smith and J. R. Clayton, Publication. Drs, Wm. Taft, A. F. Emminger and J. F. Canine, Ethics.

On motion of Dr. J. Taft it was decided to have the programmes for the next meeting made out as soon as possible so there would be ample time for the preparation of papers.

Bills amounting to eight dollars and twenty-five cents for printing and stationery were ordered paid. On motion the sum of eight dollars was voted to the janitor for his services. On motion fifteen dollars was ordered paid to the retiring secretary for his services. A motion was carried to pay the college association for fuel and gas used during the session of the society, also one to donate twenty-five dollars for refitting the college lecture room. The association then adjourned to meet on the first Friday in March, 1879, at ten o'clock a. m.

EDWARD G. BETTY,
Recording Secretary.

